# Type II NKT Cells in Inflammation, Autoimmunity, Microbial Immunity, and Cancer

**DOI:** 10.3389/fimmu.2015.00316

**Published:** 2015-06-17

**Authors:** Idania Marrero, Randle Ware, Vipin Kumar

**Affiliations:** ^1^Laboratory of Immune Regulation, Department of Medicine, University of California San Diego, La Jolla, CA, USA

**Keywords:** CD1d, innate immunity, inflammation, autoimmunity, cancer, sulfatide, lipids, neutrophils

## Abstract

Natural killer T cells (NKT) recognize self and microbial lipid antigens presented by non-polymorphic CD1d molecules. Two major NKT cell subsets, type I and II, express different types of antigen receptors (TCR) with distinct mode of CD1d/lipid recognition. Though type II NKT cells are less frequent in mice and difficult to study, they are predominant in human. One of the major subsets of type II NKT cells reactive to the self-glycolipid sulfatide is the best characterized and has been shown to induce a dominant immune regulatory mechanism that controls inflammation in autoimmunity and in anti-cancer immunity. Recently, type II NKT cells reactive to other self-glycolipids and phospholipids have been identified suggesting both promiscuous and specific TCR recognition in microbial immunity as well. Since the CD1d pathway is highly conserved, a detailed understanding of the biology and function of type II NKT cells as well as their interplay with type I NKT cells or other innate and adaptive T cells will have major implications for potential novel interventions in inflammatory and autoimmune diseases, microbial immunity, and cancer.

## Introduction

Natural killer T (NKT) cells are innate-like T cells that recognize both exogenous and endogenous lipid antigens presented by CD1d, a major histocompatibility (MHC) class I-like antigen-presenting molecule. They comprise two main subsets, type I and type II, based upon differences in the nature of their T cell receptors (TCRs) (1–3). The well-studied type I NKT cell subset that uses a semi-invariant TCRα chain is more prevalent than the type II subset in the mouse, while the less explored type II NKT cell subset that utilizes a more diverse TCR repertoire is predominant in humans (4–6). Both subsets require signaling lymphocytic activation molecule-associated protein (SAP) and promyelocytic leukemia zinc finger (PLZF) for their development and effector program (3, 7, 8). After antigenic activation, NKT cells secrete large amounts of cytokines, such as interferon-γ (IFNγ), interleukins IL-4, IL-10, IL-13, IL-17, and IL-22, tumor necrosis factor-α (TNFα), and granulocyte-macrophage colony-stimulating factor (GM-CSF), which modulate immune responses triggered by other innate NK cells and adaptive T and B cells (3–6). Both subsets appear to modulate immune responses involved in autoimmunity, inflammation, infections, and cancer (4–7, 9, 10). This review primarily focuses on lipid-reactive CD1d-restricted TCR α/β+ type II NKT cells and their potential role in immunity.

## Antigen Recognition, TCR Repertoire, and Activation of Type II NKT Cells

Type I NKT cells respond to a strong agonist α-galactosylceramide (αGalCer) as well as microbial and self-lipids. By contrast, type II NKT cells are not reactive to αGalCer and recognize self-glycolipids and self-phospholipids (5, 6, 11). A major subset of type II NKT cells recognize a naturally occurring self-glycolipid, sulfatide, which is enriched in several membranes, including myelin in the central nervous system (CNS), β-cells in the pancreas, kidney, and liver (12, 13). Recently, other self-lipids, including β-D-glucopyranosylceramide (β-GlcCer), βGalCer, lysophosphatidylethanolamine (lyso-PE), lysophosphatidylglycerol (lyso-PG), or cardiolipin and lysophosphatidylcholine (lyso-PC) have been shown to be involved in the activation of liver type II NKT cells (5, 6, 8, 12–17). Lyso-PC and β-glucosylceramide and glucosylsphingosine lipids were reported to activate human type II NKT cells as well (18, 19).

The mechanism of binding of type II NKT TCRs to antigens uses features of TCR binding shared by both type I NKT cells and conventional T cells, but also is distinct from both (12, 20, 21). Thus, type II NKT TCRs contact their ligands primarily via their β chain rather than α chain, suggesting that the TCR Vβ chain contributes significantly to antigen fine specificity (20). Sulfatide-reactive type II NKT cells express oligoclonal TCRs with a limited number of Vα- and Vβ-chains (Vα3/Vα1 and Vβ8.1/Vβ3.1). In contrast to the germline-encoded TCRα chain in type I NKT cells, only about 14% of TCR Vα and 13–27% of TCR Vβ chains in type II NKT cells are encoded by germline gene segments (12). A prevalent type II NKT cell subset expressing Vα3.2–Vβ8 TCR has also been described (22). It remains to be seen whether other type II NKT cells will also use a similar mechanism of lipid recognition to sulfatide-reactive T cells. It appears that type II NKT cells are mostly reactive to self-lipid ligands, but they can also recognize structurally similar microbial-derived lipids because of their TCR degeneracy or promiscuity.

Type I NKT cells can be activated either directly through TCR stimulation by exogenous microbial lipid antigens or indirectly by stimulatory self-lipids presented by CD1d and/or cytokines (IL-12, IL-18, or type I IFN) produced through Toll-like receptor (TLR)-mediated signaling (23, 24). Thus, different self-lipids as well as cytokines present at elevated levels during inflammation can potentially stimulate type I NKT cells. Recent studies suggest that type I NKT cells can be activated in response to bacteria, as well as viruses, without antigen receptor stimulation (25). By contrast, type II NKT cells are mainly stimulated by direct recognition of lipid/CD1d complexes by their TCR. It has been consistently found that stimulation of type II NKT hybridomas with phospholipids and glycolipids requires lipid uptake, intracellular processing, and presentation to TCR but not TLR signaling (15, 26). In many experimental conditions wherein type I NKT cells are activated, type II NKT cells remain inactivated suggesting that type II NKT cells may not be easily activated by cytokine/TLR signaling but require self-lipid recognition.

It is becoming clear that the TCR recognition by type II NKT cells can be highly specific or promiscuous. For example, sulfatide-reactive type II NKT cell hybridomas XV19 or 19.3 can recognize sulfatide or lyso-PC effectively but not so efficiently all other phospholipids or glycolipids (12–15, 17, 26). Consistent with this, at the polyclonal level, some lyso-PC-reactive NKT cells are distinct from sulfatide-reactive NKT cells (17) and in 4get mice, type II NKT cells are reactive to several self-lipids but not sulfatide (8, 16). Similarly, some lyso-PG-reactive type II NKT hybridomas can recognize both self and microbial lipids derived from *Mycobacterium tuberculosis* or *Corynebacterium glutamicum* and others are non-responsive to these lipids (15). These findings identify some redundancy as well as overlapping TCR repertoires among type II NKT cells that recognize self-lipids.

Immune regulatory activity of NKT cells can be mediated by cytokines secreted by NKT cells themselves or following their interaction with other immune cells, including DCs, NK cells, Tregs, monocytes, and B cells. Activation of NKT cell subsets can result in the deviation of a cytokine secretion profile in MHC-restricted CD4^+^/CD8^+^ T cells toward either a pronounced Th1-, Th2-, or Th17-like response.

It is noteworthy that in inflamed target tissues, such as in pancreas in non-obese diabetic (NOD) mice that spontaneously develop type 1 diabetes (T1D) and in the CNS during experimental autoimmune encephalomyelitis (EAE), both type I and type II NKT cells accumulate (13, 27). However, activation of type II NKT cells following sulfatide or lyso-PC administration leads to a rapid accumulation of type I NKT cells into liver in an IL-12 and macrophage inflammatory protein 2 (MIP2)-dependent fashion. But these recruited type I NKT cells are neither activated nor do they secrete cytokines, and consequently they are anergic, leading to decreased levels of IFNγ followed by reduced recruitment of myeloid cells, NK cells, and protection from liver damage (28, 29). In contrast to the activation of lyso-PE-reactive type II NKT cells in an infectious model of HBV, hepatic type I NKT cells are not anergized but stimulated to secrete cytokines (16). This difference in type I NKT stimulation may relate to the differential milieu in liver during sterile versus infectious immunity.

## A Novel Type II NKT Cell-Mediated Immune Regulatory Pathway

Sulfatide-mediated type II NKT cell stimulation *in vivo* results in the activation of predominantly hepatic plasmacytoid DCs (pDC) but not conventional DC (cDC) and ultimately induction of anergy in hepatic type I NKT cells. This unique immune regulatory pathway not only involves cross-regulation of type I NKT cells but also inhibition of pathogenic Th1/Th17 cells through tolerization of hepatic cDC and tissue-resident antigen-presenting cells (APCs), such as microglia in the CNS (28, 30). By contrast, activation of type I NKT cells following αGalCer administration predominantly activates hepatic cDC (28, 29). We are currently investigating the molecular mechanism of these NKT–DCs interactions.

It has been shown that this immune regulatory pathway effectively controls EAE, T1D, inflammatory liver diseases, and systemic lupus erythematosus (SLE) (17, 27, 28, 30–32) (Halder, unpublished). A recent study has suggested that the ICOS and PD-1 ligand pathways are required for the regulation of T1D in NOD mice by CD4^+^ type II NKT cells (33). Sulfatide-mediated type II NKT cell activation can also result in IL-10 secretion and, consequently, inhibition of type I NKT cells and diabetogenic or encephalitogenic CD4^+^ and CD8^+^ T cells (13, 27). Furthermore, activation of type II NKT cells also induces alterations in other innate cells, including myeloid-derived suppressor cells (MDSCs), CD11b^+^Gr-1^+^ cells, and neutrophils (17, 28, 30–32). Accordingly, MDSCs have been shown to protect mice from EAE (34). CD11b^+^Gr-1^+^ cells and neutrophil alterations can also protect from inflammatory liver diseases (31, 32, 35). Additionally, activation of type II NKT cells by PD-L1-deficient DCs increases the IL-4 and IL-13 levels and, consequently, decreases the numbers of IFNγ and IL-17-secreting pathogenic T cells (36). Thus, targeting type II NKT-mediated inhibition of the effector functions of Th1/Th17 cells and APCs in peripheral organs as well as in affected target tissues offers a potent strategy for intervention in autoimmune and inflammatory diseases (30).

## Type II NKT Cells in Autoimmune and Inflammatory Diseases

The activation of type II NKT cells with sulfatide controls both antigen-induced and spontaneously arising autoimmune diseases. Additionally, sulfatide-mediated immune regulation inhibits inflammatory liver diseases elicited by type I NKT cells (13, 17, 27, 28, 30–32, 35). Sulfatide-reactive type II NKT cells also have been shown to abrogate ischemic–reperfusion injury in mice and in patients with acute tubular necrosis (37). Interestingly, during EAE and T1D progression, sulfatide-reactive type II NKT cells accumulate in the target tissue and in the draining of lymph nodes, respectively. This greater abundance of type II NKT cells in the CNS inverts the usual ratio of type II/type I NKT cells (type II NKT cells, 3–4%, and type I NKT cells, 0.6–0.9%) (13, 27). Thus, administration of brain-derived sulfatides or synthetic cic-tetracosenoyl or tetracosenoyl sulfatide affords protection from EAE and diabetes (13, 27, 30). In both cases, it is the sulfatide with the longer fatty acid chain that is able to efficiently activate type II NKT cells and prevent autoimmunity. These data suggest that sulfatide analogs should be examined in clinical studies in multiple sclerosis (MS) and T1D. Our preliminary studies also suggest that activation of type II NKT cells following administration of sulfatide significantly inhibits development of lupus nephritis in (NZB X NZW) F1 mice, further indicating a regulatory role of type II NKT cells (5) (Halder, unpublished).

Recent studies have indicated key mutual interactions among NKT cells, CD1d^+^ cells, and commensal microbiota in the intestine (38). Evidence from several animal models of inflammatory bowel disease (IBD) demonstrates that type I NKT cells can be either protective or pathogenic (39). Interestingly, type II NKT cells seem to promote intestinal inflammation and mediate a pathogenic response when both CD1d expression and the frequency of IL-13 producing type II NKT cells are increased in mice as well as patients with ulcerative colitis (40–42). It is noteworthy that type II NKT cells involved in ulcerative colitis in humans are also reactive to lysosulfatide, but in contrast to the liver type II NKT cells, they secrete IL-13 and not IFNγ (41). This suggest that there are subsets of type II NKT cells that may have different TCR repertoires as well as different cytokine secretion patterns in different tissues, just as there are different subsets of type I NKT cells.

## Type II NKT Cells in Metabolic and Liver Disorders

Both NKT cell subsets have been shown to be involved in adipose-tissue inflammation, diet-induced obesity, and glucose metabolism (43). Roles for eosinophils, macrophages, and innate lymphoid type 2 cells (ILC2) have also been suggested in metabolic disorders (44, 45). More recently, type II NKT cells induced by both IL-25 and sulfatide treatments have been shown to be involved in the regulation of inflammation in adipose tissue and prevention of high fat diet-induced obesity in mice. Transfer of type II NKT cells into obese mice induced a greater and prolonged weight loss and improved glucose tolerance (44).

In inflammatory liver diseases, type I and type II NKT cells have been shown to play opposing roles (35). Earlier, it was shown that following liver injury after ischemic–reperfusion or ConA administration, a rapid activation of IFNγ-secreting type I but not type II NKT cells takes place (28, 31). Activation of type I NKT cells generates a cascade of events that contributes to liver inflammation and damage. The secretion of pro-inflammatory cytokines, such as IFNα, and chemokines leads to accumulation of CD11b+ Gr-1+ cells as well as other myeloid cells resulting in the destruction of hepatocytes. By contrast, sulfatide-activated type II NKT cells inhibit the cascade of pro-inflammatory events through a mechanism that includes activation of pDC resulting in tolerization of cDC, anergy in type I NKT cells and consequently protection from liver injury (28, 31, 35). In a mouse model of chronic alcohol liver disease (ALD), we have found that type I, but not type II, NKT cells are activated, leading to recruitment of inflammatory neutrophils and liver damage (46, 47). Inhibition of type I NKT cells following a novel direct mechanism involving all-trans retinoic acid (48) and its receptor (RAR-γ) signaling, or an indirect mechanism mediated by sulfatide-activated type II NKT cells significantly blunts ALD (46). Consistent with this, accumulation of activated type I NKT cells in patients with NAFLD has recently been shown (49–51). Currently, clinical studies are being carried out to examine the potential use of a RAR-γ analog for the treatment of alcohol- and non-alcoholic liver disease. We are presently investigating in humans the role of both type I and type II NKT cells in the promotion as well as regulation of inflammatory immune responses in liver and gut. The identification of the role of these cell subsets in liver disorders could potentially lead to the development of novel therapeutics.

## Type II NKT Cells in Infectious Diseases

Natural killer T cells contribute to the early immune response against a broad range of microbial pathogens, playing either a beneficial role in some infections or a negative role in others (52). Frequently, type I and type II NKT cells can have opposing roles in microbial immunity. For example, in the case of *Trypanosomacruzi* infections, type II NKT cells were shown to promote inflammation and mortality and reduced titers of pathogen-specific antibodies, whereas type I NKT cells led to reduced inflammation and improved mortality and antibody titers (53). By contrast, during murine *Schistosoma* *mansoni* infection, type II NKT cells led to increased Th2 cytokine secretion and decreased IFNγ production, while type I NKT cells reinstated IFNγ levels (54).

Sulfatide-activated type II NKT cells have also been shown to affect the course of infectious diseases. Previously, we showed that sulfatide-activated type II NKT cells inhibit HIV-1 replication and enhance multi-lineage hematopoiesis in a SCID-Hu (Thy/Liv) HIV model (55). We hypothesized that sulfatide-mediated activation of type II NKT cells and pDC results in the induction of anergy in type I NKT cells. It was shown that peripheral CD4^+^ type I NKT cells are depleted in early HIV infection and that the remaining cells in the circulation during HIV infection are functionally impaired in IFNγ expression (56). Sulfatide-mediated activation of type II NKT cells also has a protective effect in a *Staphylococcus aureus* murine model of sepsis and is associated with a decrease in pro-inflammatory cytokines, such as TNFα and IL-6 (57). This beneficial outcome was found to depend on CD1d but not on type I NKT cells.

Recent studies in HBV infection in animal models and humans have shown that NKT cells contribute to the initiation of antiviral immune responses against HBV. An early activation of type I and type II NKT cells was found following infection, mainly in the liver, which correlated with IFNγ-dependent suppression of viral replication; but also NKT cells contribute to HBV-induced hepatitis (16, 58). Using a mouse model of infection with HBV-expressing adenoviral particles (Ad-HBV), it was demonstrated that Jα18-deficient (lacking type I NKT cells) and CD1d-deficient (lacking all NKT cells) mice exhibited a significant decrease in NK, B, CD4^+^, and CD8^+^ T cell activation and hepatic immune infiltration, supporting the idea that NKT cells play a role in the immune response to HBV (16). More importantly, it was shown that HBV infection induces production of modified ER self-lipids, including phosphatidylethanolamine (PE) and lysophosphatidylethanolamine (lyso-PE), direct activation of liver type II NKT cells, and downstream cytokine-dependent activation of type I NKT cells. Type II NKT activation required hepatocyte expression of microsomal triglyceride transfer protein (MTP) and CD1d (16). In a murine acute hepatitis B transgenic model, NKG2D-dependent activation of type II NKT cells has been shown to result in liver damage (59).

## Type II NKT Cells in Tumor Immunity

Similar to the immune responses in liver, type I and type II NKT cells have been shown to play opposing roles in tumor immunity (10, 60). Type I NKT cells are usually associated with the promotion of tumor immunity, whereas type II NKT cells are associated with its suppression (61). Thus, type I NKT cells were found to induce lysis of tumor cells directly via a perforin/granzyme-dependent mechanism or indirectly by induction of Th1 cytokine secretion and activation of NK and DC cells. By contrast, type II NKT cells have shown immunosuppressive activity down-regulating tumor immunosurveillance (60, 62–64). Type II NKT cell-induced tumor suppression can be mediated by IL-13 secretion resulting in the activation of TGF-β-secreting MDSCs that inhibit tumor-specific CD8^+^ T cells or type I NKT cells. In humans, Chang et al. have also shown an increase in IL-13-secreting lyso-PC-reactive type II NKT cells in multiple myeloma patients (18). Interestingly, type I NKT cells are decreased in these patients, suggesting opposing roles, as their increased frequency is associated with better prognosis.

## Future Studies and Challenges

Availability of stable reagents for analysis of type I NKT cells has resulted in characterization of changes in their frequency and phenotype in humans in different disease conditions, including autoimmune and infectious diseases and cancer. A detailed characterization of type II NKT cell repertoires and their ligands in humans is required for a broader understanding of their physiological role in health and in disease. A recent study suggesting a role for lyso-glucosylsphingosine (lyso-GL1)-reactive type II NKT cells in Gaucher disease is an important development (19). Together, all of these observations indicate that it may be possible in the future to differentially activate or inhibit type I and type II NKT cells for the development of novel immunotherapeutic protocols in altering the course of cancer and both infectious and autoimmune diseases.

## Conflict of Interest Statement

The authors declare that the research was conducted in the absence of any commercial or financial relationships that could be construed as a potential conflict of interest.
